# A Secreted Phospholipase A_2_ Induces Formation of Smooth Muscle Foam Cells Which Transdifferentiate to Macrophage-Like State

**DOI:** 10.3390/molecules24183244

**Published:** 2019-09-06

**Authors:** Karina Cristina Giannotti, Sönke Weinert, Mariana Nascimento Viana, Elbio Leiguez, Thaís L. S. Araujo, Francisco R. M. Laurindo, Bruno Lomonte, Rüdiger Braun-Dullaeus, Catarina Teixeira

**Affiliations:** 1 Pharmacology Laboratory, Butantan Institute, 05503-900 São Paulo, Brazil (K.C.G.) (M.d.N.V.) (E.L.); 2 Department of Cardiology and Angiology, Internal medicine, Otto-von-Guericke-Universität Magdeburg, 39120 Magdeburg, Germany (S.W.) (R.B.-D.); 3 Vascular Biology Laboratory, Heart Institute (InCor), University of São Paulo School of Medicine, 01246-903 São Paulo, Brazil (T.L.S.A.) (F.R.M.L.); 4 Instituto Clodomiro Picado Institute, University of Costa Rica, 11501 San José, Costa Rica

**Keywords:** phospholipase A_2_, vascular smooth muscle cells, lipid droplets

## Abstract

Vascular smooth muscle cells (VSMCs) loaded with lipid droplets (LDs) are markers of atherosclerosis. In this disease, inflammatory Group IIA-secreted phospholipase A_2_s (GIIA sPLA_2_s) are highly expressed in VSMCs, but their actions in these cells are unknown. Here, we investigated the ability of myotoxin III (MT-III), an ophidian GIIA sPLA_2_ sharing structural and functional features with mammalian GIIA sPLA_2_s, to induce LD formation and lipid metabolism factors involved in this effect. Modulation of VSMC phenotypes by this sPLA_2_ was also evaluated. Incubation of VSMCs with MT-III significantly increased the number of LDs. MT-III upregulated scavenger receptor type 1 (SR-A1) and lectin-like oxidized low-density lipoprotein receptor-1 (LOX-1) protein expression and enhanced acetylated-low density lipoprotein (acLDL) uptake by VSMCs, revealing the ability of a GIIA PLA_2_ to modulate scavenger receptor activities. MT-III induced translocation and protein expression of PPAR-γ and -β/δ. Inhibition of peroxisome proliferator-activated receptors (PPARs) and diacylglycerol O-acyltransferase (DGAT) and acyl-CoA:cholesterolacyltransferase (ACAT) enzymes abrogated MT-III-induced LD formation. Moreover, in response to MT-III, VSMCs acquired phagocytic activity and expressed macrophage markers CD68 and MAC-2. In conclusion, MT-III is able to stimulate VSMCs and recruit factors involved in lipid uptake and metabolism, leading to the formation of VSMC-derived foam cells with acquisition of macrophage-like markers and functions.

## 1. Introduction

Vascular smooth muscle cells (VSMC) are the main stromal cells of the vascular wall and play essential roles in the regulation of blood vessel tone and blood flow [1]. Under inflammatory processes, such as atherosclerosis, in addition to the differentiation from quiescent contractile to proliferative synthetic phenotypes [2], which actively contributes to formation of atheromatous plaque, VSMCs undergo phenotypic modification that is characterized by the expression of macrophage markers and assume macrophage functions [3,4]. In addition, the differentiated VSMCs express a variety of fatty acids and cholesterol uptake receptors [5], favoring the capture of fatty acids and cholesterol, giving rise to a significant number of VSMC-derived foam cells characterized by a cytoplasm packed with lipid droplets (LDs). These cytoplasmic organelles are well-known to be found in increased numbers in cells associated with inflammatory processes, such as macrophages [6,7]. Among the proteins compartmentalized by LDs are those from the perilipin family (PLIN). These proteins are recruited to the ER during LD biogenesis, favoring fatty acids capture [8] and regulating LD formation [9]. LDs are composed mainly of triacylglycerol and cholesterol. The production of these neutral lipids is regulated by the enzymes diacylglycerol O-acyltransferase (DGAT) and acyl-CoA:cholesterolacyltransferase (ACAT), which esterify triacylglycerol and cholesterol, respectively, leading to the storage of these neutral lipids in LDs [10,11]. In addition, the transcription factors peroxisome proliferator-activated receptors (PPARs) orchestrate LD biogenesis through free fatty acid uptake by increasing the expression of scavenger receptors and proteins related to lipid metabolism [12]. 

Secreted PLA_2_s (sPLA_2_s) are recognized as key regulators of LD formation by providing free fatty acids from membrane phospholipids that are essential for this process, thus directly regulating the assembly of these organelles [13]. This superfamily of enzymes comprises several groups, including those of group IIA (GIIA), which have been reported to be expressed in high levels in vessel wall cells, such as VSMCs, in macrophage-rich regions, and in the extracellular matrices of the affected intima during the atherosclerotic process [14]. Presently, the participation of GIIA sPLA_2_s in the development of atherosclerosis is associated with their ability to modify low-density lipoprotein (LDL) particles, generating more proaterogenic particles and the production of inflammatory lipid mediators [15]. However, the potential role of this group of enzymes in the formation of VSMC-derived foam cells needs to be addressed.

Snake venom group IIA sPLA_2_s share structural and functional features with mammalian inflammatory group IIA sPLA_2_s [16]. Similar to mammalian sPLA_2_s, the venom sPLA_2_s, such as MT-III, which has been isolated from *Bothrops asper* snake venom, exhibit the ability to induce inflammatory events in both in vivo and in vitro experimental models [17,18,19]. Considering the contribution of sPLA_2_s to lipid accumulation and the formation of LDs in macrophages, the question arises of the effect MT-III has on VSMC regarding the formation of LD formation, considering related mechanisms and the phenotypical identity of VSMCs. 

## 2. Results

### 2.1. LD Formation Induced by MT-III in Vascular Smooth Muscle Cells

**Figure 1:**
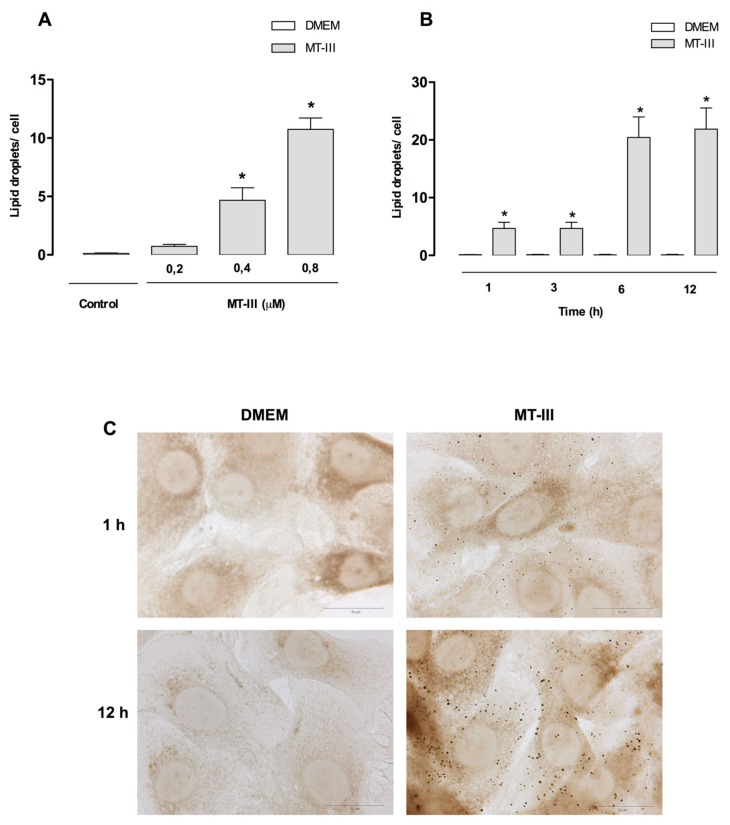
MT-III induces LD formation in VSMCs. (**A**) Effect of MT-III on LD formation in VSMCs stimulated with selected concentrations of MT-III or with DMEM (control) for 1 h. (**B**) Time-course of MT-III-induced LD formation. VSMCs (8 × 10^3^ cells/coverslip) were incubated with MT-III (0.4 µM) or DMEM (control) for 1, 3, 6, or 12 h. LDs were quantified using light microscopy after OsO_4_ staining. (**C**) Osmium-stained LDs observed in control or MT-III (0.4 µM) stimulated cells for 1 or 12 h. Each bar represents the mean ± SEM of the number of LDs/cell in 50 cells. Values represent mean ± SEM for six animals (*n* = 6) (ANOVA).Note: * *p* < 0.05 compared with vehicle treated cells.

To access the capability of a GIIA sPLA_2_ MT-III to induce LD formation in VSMC, cells were incubated with selected concentrations of MT-III. Preliminary assays were performed using VSMCs freshly isolated from rat aorta (passage zero) to evaluate the influence of differentiation state of cells and cell phenotypes on lipid droplets formation induced by MT-III. The incubation of MT-III (0.4 and 0.8 µM) for 3, 6, and 12 h did not increase lipid droplet formation in comparison with non-stimulated control cells (data not shown). Therefore, in the present study, modulation and de-differentiation of VSMCs was induced by serum before the addition of MT-III and all assays were performed using the 5th to 8th passage cells. In this experimental condition VSMCs are responsive to MT-III stimulus, producing lipid droplets. As demonstrated in Figure 1a,b, incubation of VSMC with MT-III induced a significant increase of LDs in a time- and concentration-dependent manner. MT-III incubated for 1 h at concentrations from 0.4 to 0.8 µM induced a significant increase in the number of LDs in comparison with control cells incubated with culture medium alone. This effect was detected up to 12 h (Figure 1b). As illustrated in Figure 1c, control VSMCs stained with OsO_4_ showed no LDs in the cytoplasm. In contrast, MT-III-stimulated VSMCs exhibited a cytoplasm packed with the osmiophilic organelles, which can be seen as dark punctate structures. These data indicate that MT-III induces LD formation in VSMC in a time- and dose-dependent manner. 

### 2.2. PLIN2 and PLIN3 Co-Localize with LDs in MT-III-Stimulated VSMCs

**Figure 2:**
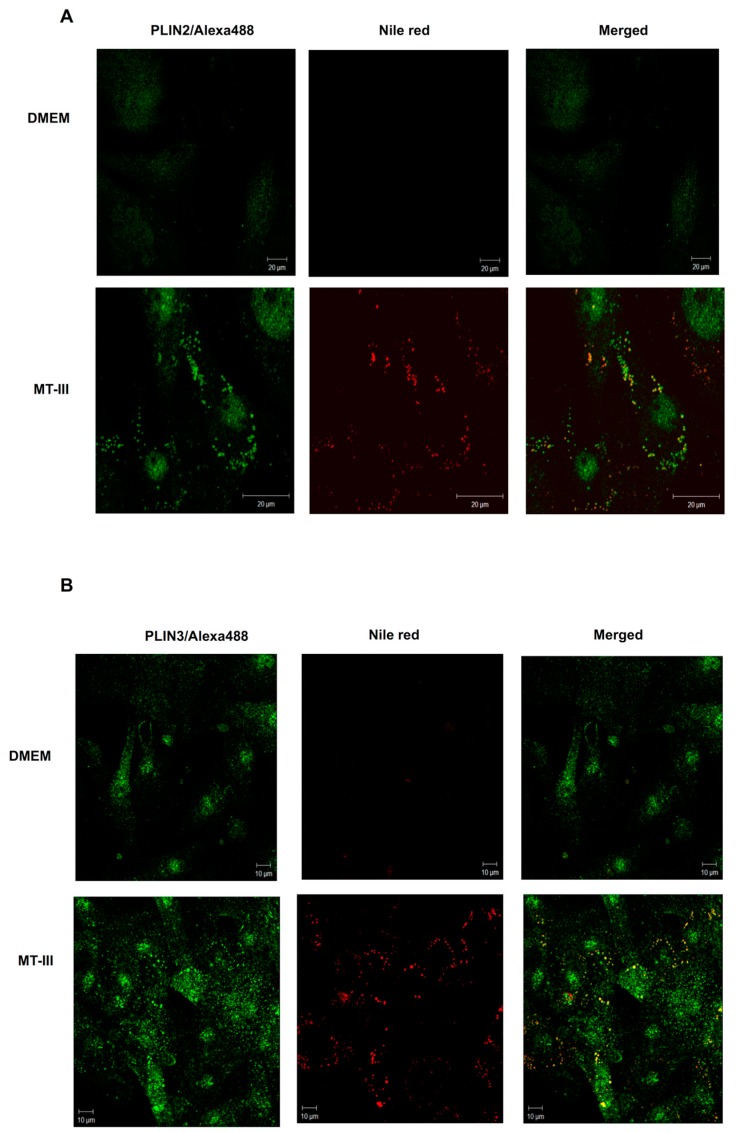
MT-III induces PLIN2 and PLIN3 recruitment to LDs in VSMCs. VSMCs (8 × 10^3^ cells/coverslip) were incubated with DMEM (control) or MT-III (0.4 µM) for 12 h and labelled for LDs (with Nile red) and for PLIN2 (**A**) or PLIN3 (**B**) (with fluorescein isothiocyanate- FITC-conjugated secondary antibody). The merged image shows co-localization of PLIN2 and PLIN3 to LDs. The pictures are representative of three independent experiments (*n* = 9).

**Figure 3:**
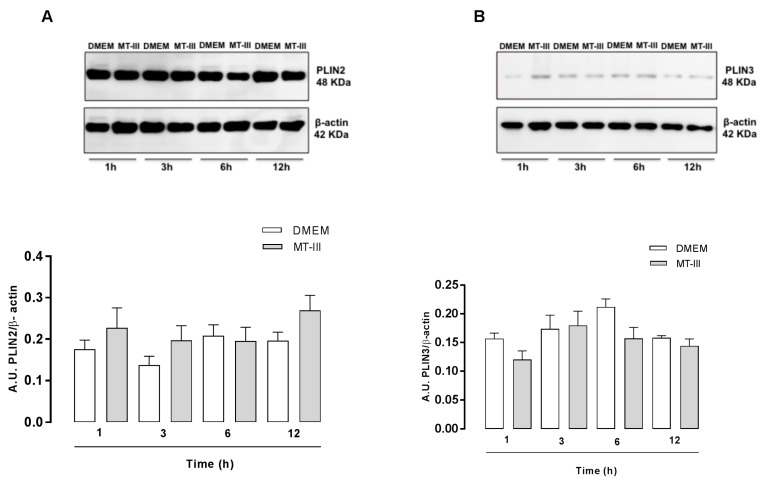
MT-III does not affect PLIN2 or PLIN3 protein expression in VSMCs. VSMCs (5 × 10^4^ cells/well) were incubated with DMEM (control) or MT-III (0.4 µM) for 1, 3, 6, and 12 h. Western blot analysis of PLIN2 (**A**) and PLIN3 (**B**) and β-actin (loading control) of the VSMC extracts and the densitometric analysis (in arbitrary units - A.U.) of PLIN2 and PLIN3 expression normalized to β-actin. The results are expressed as the mean ± SEM for three independent experiments (*n* = 9) (ANOVA).

To better understand the stimulatory effect of MT-III on VSMCs leading to LD formation, cells exposed to MT-III were immunostained with specific antibodies that recognize either the scaffold proteins for LD assembly PLIN2 or PLIN3 and counterstained for neutral lipids of the LD core using Nile red. As illustrated in Figure 2a,b, VSMCs stimulated with MT-III (0.4 µM) for 6 h exhibited strong fluorescent staining (green) for PLIN2 and PLIN3, with a punctate cytoplasmic pattern that was absent in the non-stimulated control cells. Fluorescent Nile Red-labelled LDs were virtually absent in non-stimulated control VSMCs. In VSMCs incubated with MT-III, cytoplasmic-stained PLIN2 and PLIN3 co-localized with Nile red-labelled neutral lipid inclusions. No significant staining was detected in control VSMCs. In a further set of experiments, we investigated whether MT-III would induce the protein expression of these LD structural proteins. Western blotting analysis revealed no changes in the levels of PLIN2 and PLIN3 protein expression in the extracts of cells stimulated with MT-III at any of the incubation times tested (Figure 3a,b). Altogether, these data reveal that MT-III is able to induce the recruitment of PLIN2 and PLIN3 from their constitutive sites and confirm the role of these LD scaffold proteins upon stimulation by the sPLA_2_ MT-III.

### 2.3. Acyl CoA: Diacylglycerolacyltransferase (DGAT) and Acyl-CoA:Cholesterolacyltransferase (ACAT) Contribute to LD Formation Induced by MT-III

**Figure 4:**
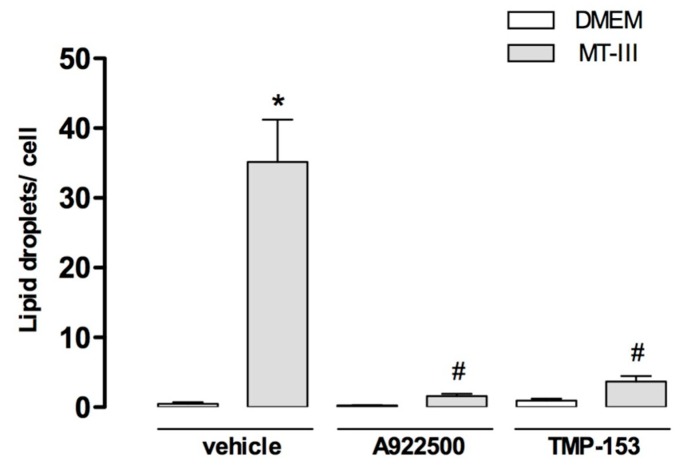
Effect of DGAT and ACAT inhibitors on LD formation induced by MT-III. VSMCs (5 × 10^3^ cells/well) were incubated with A922500 (100 nM) or TMP-153 (100 nM) or vehicle (< 1% DMSO or RPMI) for 1 h and then with MT-III (0.4 μM) for 12 h. LDs were quantified using light microscopy after osmium staining. Each bar represents the mean ± SEM LDs/cell in 50 counted cells. Values represent means ± SEM from three independent experiments (*n* = 9) (ANOVA). Note: * *p* < 0.05 compared with vehicle treated cells; ^#^ *p* < 0.05 compared with vehicle treated cells stimulated with MT-III.

Neutral lipids are synthesized locally at LDs during lipid loading [20,21]. As demonstrated in Figure 4, the pre-treatment of cells with A922500, a DGAT inhibitor, abolished MT-III-dependent LD formation. Additionally, the treatment of cells with TMP-153, an ACAT inhibitor, caused a reduction of 77.6% in the number of LDs in MT-III-stimulated VSMCs compared with vehicle-treated cells stimulated with MT-III. Altogether, these findings show that enzymes involved in the synthesis of neutral lipids, such as triacylglycerol and cholesterol, are implicated in LD formation induced by MT-III. 

### 2.4. MT-III Induces Increased Protein Expression of ABC Lipid Transporters 

**Figure 5:**
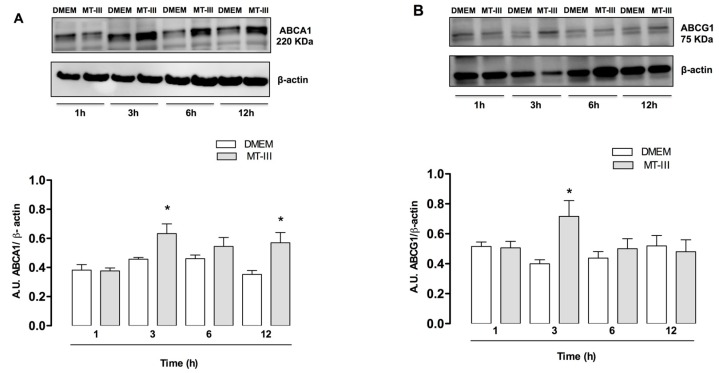
ABCA1 and ABCG1 are upregulated in VSMCs incubated with MT-III. VSMCs (5 × 10^4^ cells/well) were incubated with MT-III (0.4 μM) or DMEM (control) for 1, 3, 6, and 12 h. (**A**) Western blotting and densitometric analysis of ABCA1 (**A**) and ABCG1 (**B**) and β-actin (loading control) in VSMC extracts. The densities were normalized with those of β-actin. The results are expressed as the mean ± SEM from three experiments (*n* = 9) (ANOVA). Note: * *p* < 0.05 compared with control cells.

ATP-binding cassette transporters ABCA1 and ABCG1 play a major role in cholesterol efflux of macrophage-derived foam cells [22]. As demonstrated in Figure 5a, MT-III induced a significant increase in ABCA1 protein expression at 3 and 12 h after stimulation with MT-III in comparison with control cells. In addition, increased expression of ABCG1 was also observed after 3 h of stimulation with MT-III returning to the basal levels after this time of incubation (Figure 5b). These data indicate that MT-III upregulates ABCA1 and ABCG1 in VSMC.

### 2.5. MT-III Induces Protein Expression and Translocation of PPAR-γ and PPAR-δ/β in VSMCs

**Figure 6:**
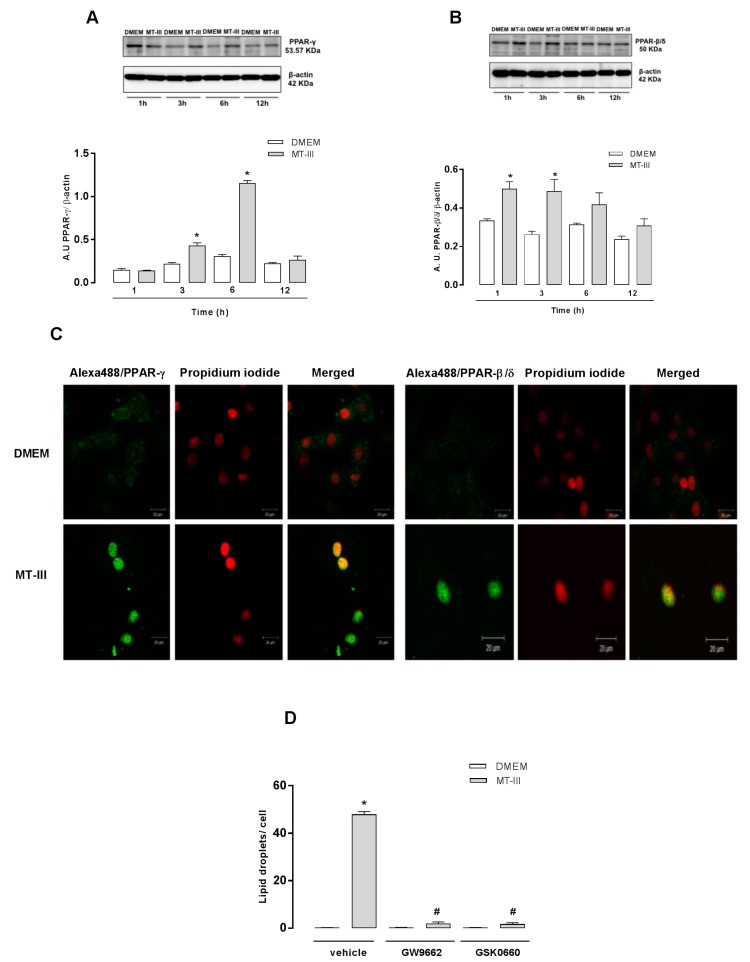
MT-III induces activation and upregulation of PPAR-γ and PPAR-δ/β expression in VSMCs. VSMCs (5 × 10^4^ cells/well) were stimulated with MT-III (0.4μM) or DMEM (control) for 1, 3, 6, and 12 h. (**A**,**B**) Western blotting and densitometric analysis of PPAR-γ or PPAR-δ/β and β-actin (loading control) in VSMC extracts. The densities were normalized with those of β-actin. (**C**) VSMCs (8 × 10^3^ cells/coverslip) incubated with DMEM (control) or MT-III (0.4 μM) for 1 or 3 h were labeled with anti-PPAR-γ or PPAR-δ/β and visualized with a secondary antibody conjugated to Alexa488 (green). The nucleus was stained with iodide propidium. The pictures prove an activation and translocation of the PPARs to the nucleus. Shown pictures are representative of three independent experiments. (**D**) VSMCs (8 × 10^3^ cells/coverslip) were incubated with GW9662 or GSK0660 (10 μM) PPAR inhibitors for 1 h and then stimulated with MT-III (0.4 μM) for 12 h. LDs were quantified using light microscopy subsequent to osmium staining. Each bar represents the mean ± SEM LDs/cell in 50 counted cells from three independent experiments (*n* = 9) (ANOVA). Note: * *p* < 0.05 compared with vehicle treated cells; ^#^ *p* < 0.05 compared with vehicle treated cells incubated with MT-III.

Considering the ability of PPAR receptors to regulate genes involved in lipid homeostasis and inflammation [23], we investigated whether MT-III would induce the protein expression and translocation of these transcription factors in VSMCs. Western blotting analysis revealed the increased expression of PPAR-γ at 3 and 6 h and PPAR-β/δ at 1 and 3 h after incubation with MT-III (Figure 6a,b). In control cells, these receptors were expressed on a basal level. To confirm the translocation of these factors after MT-III stimulation in VSMCs, an immunofluorescence staining was performed. As illustrated in Figure 6c, immunofluorescence microscopy revealed that VSMCs stimulated with DMEM (control) for 6 h exhibited fluorescent staining (green) for PPAR-γ, which did not co-localize to nucleus staining (red). However, in VSMCs stimulated with MT-III (0.4 μM) for 6 h, fluorescent labeling for PPAR-γ was shown to be co-localized to the nucleus, indicating translocation of this factor into the nucleus as a result of MT-III stimulation. Similarly, when VSMCs were incubated with MT-III (0.4 µM) for 1 h and stained for PPAR-β/δ, an identical localization in the nucleus was observed. To verify the importance of PPARs in MT-III-induced LD formation, cells were pre-treated with GSK0660, a PPAR-β/δ inhibitor, and with GW9660, a PPAR-γ inhibitor. As shown in Figure 6d, pre-treatment of cells with either PPAR-β/δ or PPAR-γ inhibitor abolished LD formation induced by MT-III. These data reinforce the relevant role of PPARs in the LD formation induced by MT-III.

### 2.6. MT-III Upregulates SR-A1 and LOX-1 But Not CD36 Protein Expression. None of These Receptors Are Involved in Lipid Accumulation Induced by MT-III

**Figure 7:**
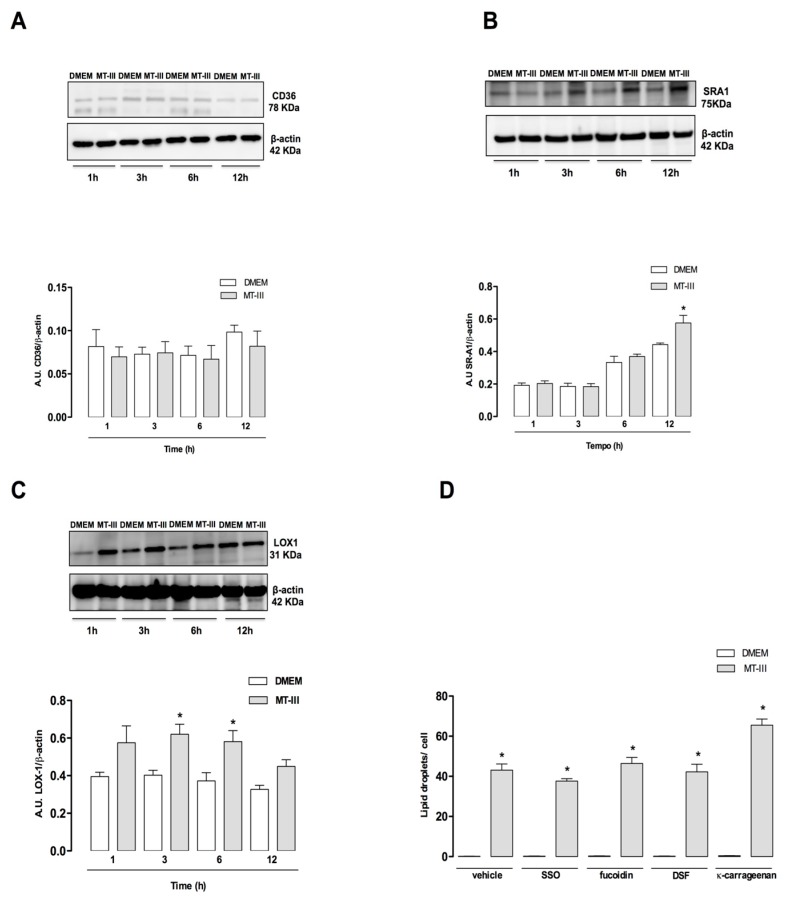
Effect of MT-III on protein expression of SR-A1, LOX-1, and CD36 by VSMCs and the effect of scavenger receptor inhibitors on LD formation induced by MT-III. VSMCs (5 × 10^4^ cells/well) were incubated with MT-III (0.4 μM) or DMEM (control) for 1, 3, 6, and 12 h. Western blotting and densitometric analysis of CD36 (**A**), SR-A1 (**B**), LOX-1 (**C**), and β-actin (loading control) in VSMC extracts. The densities were normalized with those of β-actin. (**D**) Treatment of VSMCs (8 × 10^3^ cells/coverslip) with SSO (250 μM) or fucoidin, dextran sulphate sodium salt (250 μM), or kappa-carrageenan (250 μM) for 1 h before stimulation with MT-III (0.4 µM) for 12 h. LDs were counted using light microscopy after osmium staining. The results are expressed as the mean ± SEM from three independent experiments (*n* = 9) (ANOVA). Note: * *p* < 0.05 compared with control cells.

Foam cell formation, in general, is related to the activation of scavenger receptors, such as CD36, SR-A1, and LOX-1. As demonstrated in Figure 7a, MT-III did not affect the CD36 protein content, but it induced a significant increase in SR-A1 protein expression at 12 h after stimulation in comparison with control cells (Figure 7b). In addition, MT-III caused a significant increase in LOX-1 protein expression after 3 and 6 h of stimulation. CD36, SR-A1, and LOX-1 were minimally expressed or absent in control non-stimulated VSMCs (Figure 7a–c). To verify the participation of these receptors in the MT-III induced LD accumulation, VSMCs were incubated with compound sulfo-N-succinimidyl oleate (SSO), an inhibitor of CD36, kappa-carrageenan, a ligand of LOX-1, or fucoidin or dextran sulphate sodium salts, ligands of SR-A1. As demonstrated in Figure 7d, none of the inhibitors reduced the number of LDs in MT-III-stimulated VSMCs compared with vehicle-treated VSMCs stimulated with MT-III, demonstrating that MT-III induced LD accumulation is independent of scavenger receptor function. 

### 2.7. MT-III Increases acLDL Uptake by VSMCs

**Figure 8:**
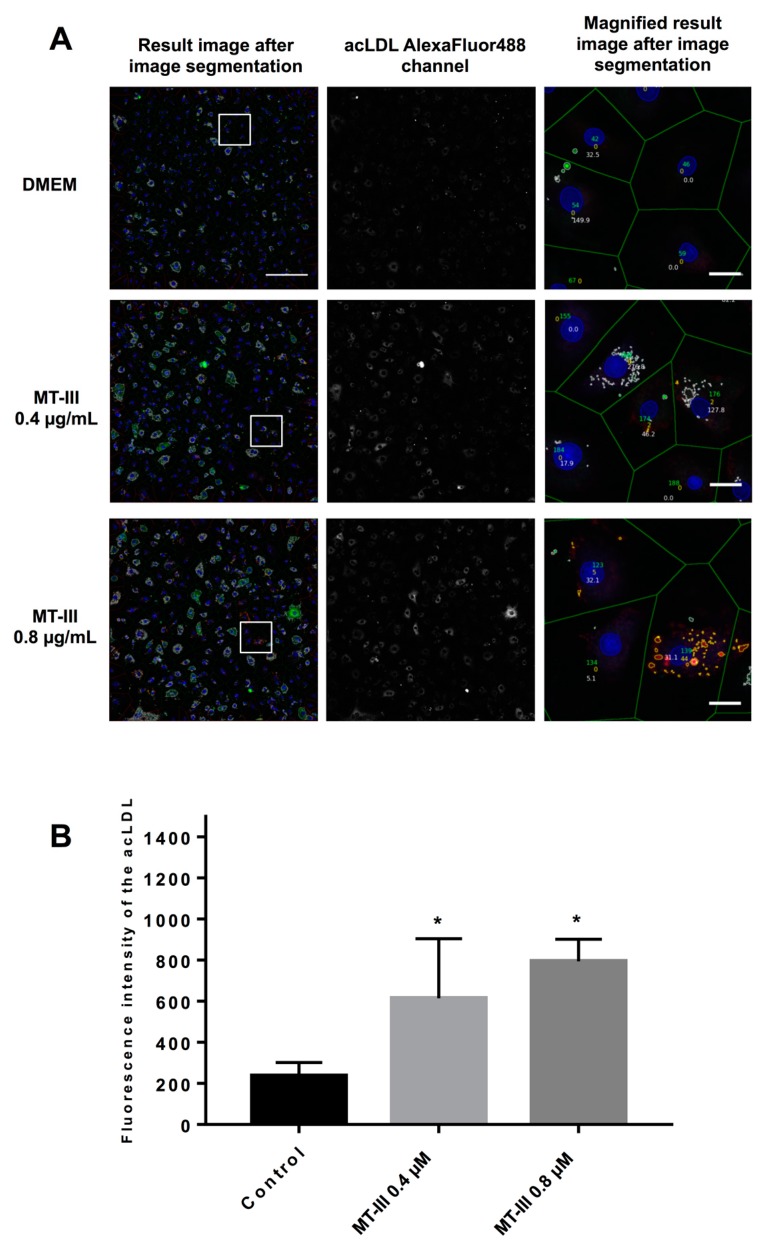
Effect of MT-III on acLDL uptake by VSMCs. (**A**) The first column illustrates the result image from the high content analysis made with the CellProfiler (scale bar 250 µM). The second column illustrates the single channel of the incorporated AlexFluor488 labeled acLDL. The third column shows a magnified view of the result image (Scale bar 25 µM). (**B**) Quantification of the number of Alexa Fluor 488-acLDL particles captured by VSMCs upon stimulation by the sPLA_2_ MT-III. The results are expressed as the mean ± SEM from two independent experiments (*n* = 6) (ANOVA). Note: * *p* < 0.05 compared with control cells.

The role of SR-A1 and LOX-1 receptors in the uptake of modified lipids has been largely evidenced [24,25]. On this basis, the ability of MT-III to modulate acLDL uptake in VSMCs was further investigated by treating cells with MT-III for 12 h followed by exposure to acLDL (1.5 µg/mL) for 24 h. As shown in Figure 8a,b, fluorescent acLDL particles were virtually absent in non-stimulated control VSMCs. After incubation with MT-III (0.4 and 0.8 μM), significant increase in the uptake of Alexa Fluor 488-acLDL by VSMCs was seen in comparison with control cells. These data reveal that MT-III exerts stimulatory effects on SR-A-1-mediated acLDL uptake activities. Moreover, despite the reported low affinity of LOX-1 receptor to acLDL uptake [26], the contribution of this receptor to the effect triggered by MT-III cannot be ruled out.

### 2.8. Macropinocytosis But Not Receptor-Mediated Endocytosis is Involved in MT-III-Induced LDs Formation

**Figure 9:**
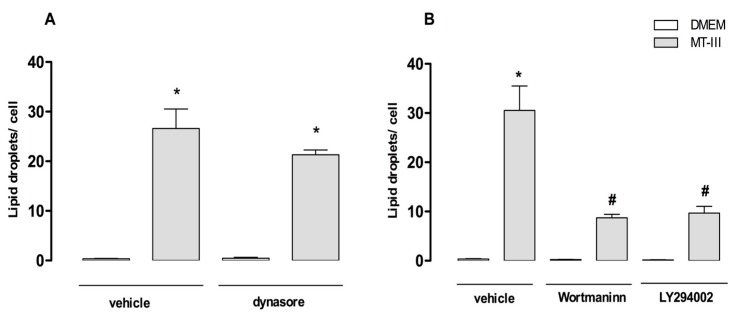
Effect of inhibitors of macropinocytosis and receptor-mediated endocytosis in MT-III-induced LD formation in VSMCs. VSMCs (8 × 10^3^ cells/coverslip) were incubated with one of the following inhibitors before stimulation with MT-III (0.4 µM) for 12 h: (**A**) dynasore (100 µM) for 1 h or (**B**) LY294002 (100 µM) or wortmaninn (2 µM) for 1 h. LDs were counted using light microscopy after osmium staining. Each bar represents the mean ± SEM of the number of LDs/cell in 50 cells. Values represent means ± SEM for three independent experiments (*n* = 9) (ANOVA). Note: * *p* < 0.05 compared with vehicle treated cells; # *p* < 0.05 compared with vehicle treated cells incubated with MT-III.

To verify the role of endocytosis in MT-III-induced LD formation, VSMCs were pre-treated with dynasore (100 μM), an inhibitor of dynamin cytoskeleton protein known to block receptor-mediated endocytosis, or its vehicle (control) before incubation with MT-III (0.4 μM), followed by the quantification of osmium-stained LDs. As demonstrated in Figure 9a, dynasore did not change the number of LDs in MT-III-stimulated VSMCs compared with vehicle-treated VSMCs. Recently, many studies have demonstrated that lipid uptake by VSMCs also occurs through the macropinocytosis process, which is dependent on PI3K [27,28]. In this sense, PI3K inhibitors are considered classical inhibitors of this process. To verify the role of macropinocytosis in MT-III induced LD formation, VSMCs were pre-treated with inhibitors of PI3K, wortmaninn (2 μM), or LY294002 compound (100 μM) or their vehicles for 1 h. Next, cells were incubated with MT-III (0.4 μM) or DMEM for 12 h. As shown in Figure 9b, treatment of VSMCs with either wortmaninn or LY294002 caused a significant reduction in the number of LDs in MT-III-stimulated VSMCs compared with vehicle-treated and MT-III-stimulated cells (70 and 60.8%, respectively). These findings indicate that macropinocytosis is implicated in LD formation induced by MT-III.

### 2.9. MT-III Increases VSMC Phagocytic Activity and Upregulates Macrophage-Related Genes

**Figure 10:**
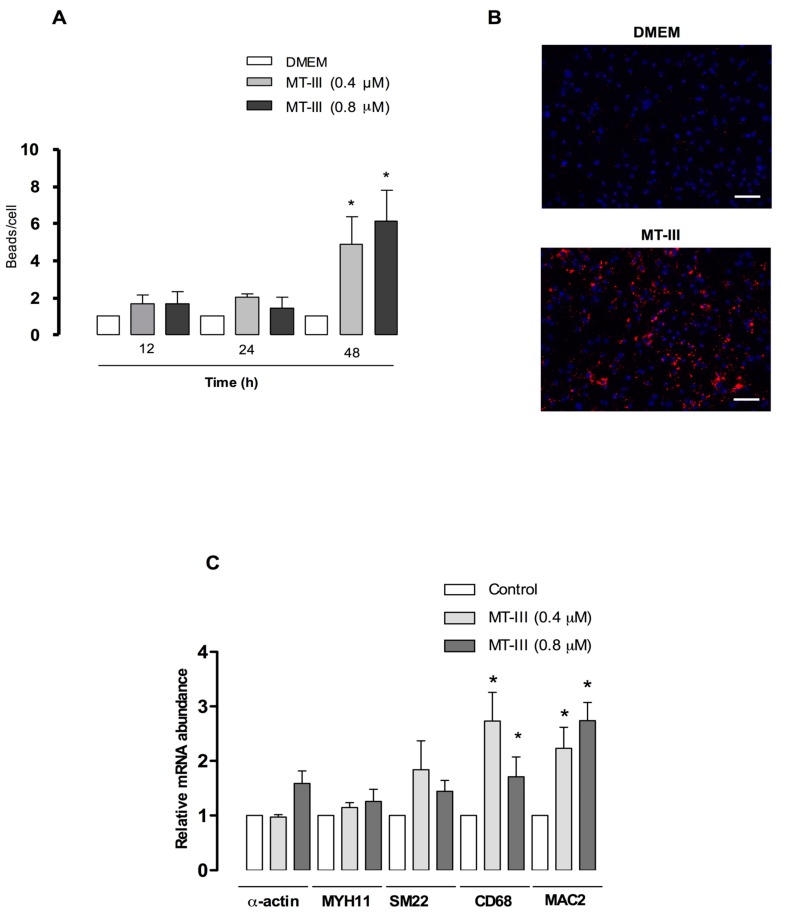
Phagocytotic activity and upregulation of macrophage markers in VSMCs induced by MT-III. VSMCs (2.5 × 10^3^ cells/well) were treated with MT-III (0.4 and 0.8 μM) or DMEM (Control) for 12, 24, and 48 h, followed by incubation with 0.5 μm fluorescent beads (red) for 6 h. Cells were then washed extensively, fixed with paraformaldehyde (4%), counterstained with Hoechst 33342 (blue-nuclei), and analyzed with an automated Axiovert 200 m followed by a computerized automatic quantification in the CellProfiler analysis software. (**A**) The number of latex beads per cell was counted to obtain numerical data for the total phagocytotic activity. (**B**) A representative field of view. (**C**) The effect of MT-III on VSMC and macrophage-related gene expression in rat VSMCs. Subconfluent rat VSMCs (8 × 10^5^ cells/well) were treated with MT-III (0.4 and 0.8 μM) or with DMEM medium (control) for 48 h. Total RNA was extracted and subjected to RT-PCR analysis. Values represent the mean ± SEM from six animals (*n* = 6) (ANOVA). Note: * *p* < 0.05 compared with control cells.

To investigate whether MT-III would also induce VSMCs to acquire functional aspects of macrophages, such as phagocytosis, VSMCs were incubated with MT-III (0.4 or 0.8 μM) for 12, 24, or 48 h followed by incubation with 0.5-μm fluorescent beads for 6 h. As shown in Figure 10a, control VSMCs presented low phagocytotic activity (1 bead/cell). After incubation with MT-III (0.4 and 0.8 μM) for 48 h, the accumulation of latex beads per cell significantly increased (489 and 613%, respectively) in comparison with controls. No significant changes in the phagocytic activity of VSMCs were seen at 12 or 24 h of incubation. Figure 10b shows VSMCs incubated with MT-III for 48 h. In control cells, a negligible number of beads per cell was seen. However, the incubation of VSMCs with MT-III for 48 h led to increased phagocytic activity and consequently an accumulation of beads in these cells. The ability of VSMCs to sustain phenotypic modulation is a result of changes in VSMC gene expression [29]. Therefore, we evaluated if MT-III would modulate the rat VSMC genes (smooth muscle alpha actin, SM22 alpha, and MHY11), driving them into a phenotype that is positive for macrophage markers (CD163, CD68, and MAC2). For this purpose, VSMCs were incubated with MT-III for 48 h and processed for gene expression analysis. Figure 10c demonstrates that the incubation of VSMCs with MT-III at concentrations of 0.4 and 0.8 μM induced an increased expression of the typical macrophage genes CD68 and MAC2, which was significantly different from control cells (223%, 92% and 274%, 273%, respectively). No alteration was seen in the gene expression of the VSMC markers alpha actin, SM22α, or MYH1 during the analyzed period. These findings demonstrate that MT-III-stimulated VSMCs acquire the macrophage phenotype with phagocytic activity. 

## 3. Discussion

In this study, we present data demonstrating, for the first time, the ability of MT-III, a GIIA sPLA_2_, to induce the formation of LDs in VSMCs and dissect the lipid metabolism factors involved in this effect. In addition, the ability of sPLA_2_s to induce VSMC phenotypic switch to macrophage-like cells is also reported, to the best of our knowledge for the first time. Our results also show that the MT-III-induced formation of lipid droplets in VSMCs is a concentration- and time-dependent effect. The ability of GIIA and GX sPLA_2_s to induce the formation of LDs and macrophage-derived foam cells has been previously demonstrated [13,30,31].

PLIN2 and PLIN3 are considered scaffold proteins for LD formation. During lipid loading, PLIN2 is mobilized from the cell membrane to the cytoplasm and contributes to long-chain fatty acid capture and LD formation [32]. PLIN3—which is constitutively found in the cytoplasm of cells—acts in the same way, contributing to LD formation and foam cell formation [33]. In this context, our findings, demonstrating that MT-III recruits PLIN2 and PLIN3 from their constitutive storages, imply the importance of both proteins for the LD assembly in VSMCs. 

Triacylglycerol (TG) and cholesterol (CE) are major components of LDs and are synthesized by DGAT and ACAT enzymes, which are located in the endoplasmic reticulum [23,24]. The contribution of the ACAT enzyme to VSMC-derived foam cell formation has already been reported in stimulated mouse VSMCs by cholesterol-cyclodextrin complexes [29]. Our results showing that the inhibition of both DGAT and ACAT enzymes abolished the LD formation induced by MT-III implicate these enzymes in the mechanism involved in MT-III-induced LD formation. Their contribution probably occurs due to their ability to provide TG (triacylglycerol) and cholesteryl esters (CE) to LD assembly. ABCA1 and ABCG1 are two known common regulators of intracellular fatty acid levels and play an important role in regulating foam cell formation. The former plays a critical role in the efflux of free cholesterol from cells [25] by removing this lipid from the cell cytoplasm to lipid-poor apoA1 particles, and the latter acts in concert with ABCA1, being responsible for cellular cholesterol efflux to lipidated high-density lipoprotein (HDL) [25,34]. In addition, during pathophysiological conditions after initial cholesterol loading, VSMCs upregulate ABCA1 and ABCG1 levels. With continued lipid loading, VSMCs downregulate the levels of the cholesterol transporters, enhancing the formation of foam cells [35]. In this sense, our results demonstrating increased protein expression of both ABCA1 and ABCG1 upon stimulus by MT-III are in line with the accumulation of high levels of lipids induced by MT-III, suggesting a mechanism by which VSMCs restore the lipid balance of homeostasis after the formation of LDs. In line with our hypothesis, the ability of 17β-estradiol to promote cholesterol efflux from VSMCs through a mechanism dependent upon the upregulation of ABCA1 and ABCG1 has been described [36]. 

It is known that fatty acid metabolism is regulated by many transcription factors, such as PPARs. During atherosclerosis, PPARs display dual roles as they can contribute to either progression or resolution of the inflammatory process and foam cell formation [37]. These transcription factors can increase lipid uptake by human VSMCs through increased expression of not only PLIN2, but also by the scavenger receptors SR-A1 and CD36, which are related to fatty acid capture and foam cell formation [38]. Our results demonstrating that MT-III upregulated PPAR-γ and β/δ protein expression in VSMCs and that the pharmacological inhibition of both isoforms abolished the LD formation induced by MT-III indicate the essential role of these receptors in the formation of LDs upon stimulation by this sPLA_2_ in VSMCs. In contrast to these findings, although the upregulation of SR-A1 and LOX-1 expression was found in MT-III-stimulated VSMCs, scavenger receptors, including CD36, do not play a role in the LD accumulation induced by MT-III, as demonstrated by receptor- specific pharmacological interventions. In spite of these data, increased expression of LOX-1 and SR-A1 scavenger receptors was translated into a marked uptake of acLDL by VSMCs stimulated with MT-III. These findings demonstrate for the first time that a GIIA PLA_2_ is capable of modulating the capture of modified lipids through increased expression of scavenger receptors, such as SR-A1 and LOX-1. Considering these results, the increased expression of the efflux transporters ABCA1 and ABCG1 found in MT-III-stimulated cells may be a consequence of the increased uptake of acLD and other fatty acids to restore lipid balance of MT-III-stimulated cells. 

Macropinocytosis is recognized as an alternative pathway by which cells can accumulate lipids, especially lipids that have undergone minimal or no chemical modifications, and has been described as an essential process for VSMC-derived foam cell formation [27,28]. Our results provide the first evidence that macropinocytosis contributes to the LD formation induced by a sPLA_2_, demonstrating that the inhibition of PI3K, a signaling protein essential for the macropinocytosis process, results in a marked reduction of the LD formation induced by MT-III. However, the mechanism by which MT-III activates PI3K was not investigated. On the other hand, the process of endocytosis mediated by the receptor is not involved in the LD formation induced by this sPLA_2_. 

Recently, a number of reports have demonstrated that a large proportion of foam cells found in atherosclerotic lesions are derived from VSMCs rather than monocytes [39]. In addition, the expression of the macrophage markers, such as CD68, MAC2, and CD163 [29,34], by foam VSMCs and the presence of cells expressing both VSMCs and macrophage markers has been demonstrated in human aortic intima preparations [39]. Moreover, VSMCs loaded with lipids have been shown to acquire the macrophage phenotype, with phagocytic activity and the capability to express macrophage markers. Our finding that MT-III-stimulated VSMCs were capable of phagocytosis indicate that MT-III induces a gain of phagocytic activity by VSMCs. This phenomenon was accompanied by the increased expression of the macrophage markers MAC2 and CD68, providing evidence that MT-III induces the transdifferentiation of VSMCs into a macrophage-like phenotype. Our data are in agreement with the existing literature demonstrating that VSMC-derived foam cells are able to perform macrophage functions [29,40]. Although the mechanisms by which MT-III modulates VSMC plasticity remain to be clarified, this is to the best of our knowledge the first demonstration of the ability of a sPLA_2_ to induce modulation of the VSMC phenotype.

In summary, the data obtained herein allow us to conclude that MT-III induces LD formation in VSMCs. This phenomenon was shown to be dependent on the enzymes ACAT and DGAT, PPAR-γ and β/δ, and the macropinocytosis process, but not on the scavenger receptors CD36, SR-A1, or LOX-1. Furthermore, MT-III is capable of inducing phenotypic modulation in these cells by reprogramming them at a transcriptional level to a different functional behavior by acquiring macrophage-like characteristics. Finally, our study provides new insights into the GIIA-secreted phospholipase A_2_ activities in VSMCs and the role of this class of enzymes in VSMC-derived foam cell formation, as well as in their phenotypic plasticity. 

## 4. Material and Methods 

### 4.1. Animals

Male Wistar rats (180–220 g) were obtained from the Butantan Institute, São Paulo, Brazil. Animals were housed in a temperature-controlled room (22–24 °C) with a 12 h light–dark cycle and fresh water and food ad libitum until use. Animals were euthanized after anesthesia by intraperitoneal (ip) injection of pentobarbital (100 mg/kg). The study was approved by the Butantan Institute Animal Experimentation Ethics Committee (reference no. 1024/13) and all procedures were in accordance with the NIH Guideline for the Care and Use of Laboratory Animals (NIH Publication, 8th Edition, 2011).

### 4.2. Phospholipase A_2_

Group IIA sPLA_2_ from *B. asper* venom, named MT-III (Uniprot accession no: P20474), was purified by ion-exchange chromatography on CM-Sephadex C-25 using a KCl gradient from 0 to 0.75 M at pH 7.0, as previously described [41], followed by RP-HPLC on a semi-preparative C8 column (Vydac; 10 × 250 mm, 5 μm particle size) eluted at a flow rate of 2.5 mL/min with a gradient of acetonitrile (0–70%, containing 0.1% trifluoroacetic acid) over 30 min [42]. Homogeneity was verified by SDS-PAGE run under reducing conditions, in which a single band of 14 kDa was observed. The complete amino acid sequence of this enzyme has been described previously [41]. The absence of endotoxin contamination in the MT-III batches used was demonstrated by performing a quantitative LAL test, which revealed undetectable levels of endotoxin (<0.125 EU/mL).

### 4.3. Cell Isolation and Culture

Rat VSMCs were obtained by spontaneous outgrowth from the thoracic aorta, as described by Metz el al. [43]. In brief, the thoracic aorta was dissected and transferred to a Petri dish with phosphate buffered saline (PBS) pH 7.2 supplemented with 40 µg/mL gentamicin sulphate (Thermo Fischer, Waltham, MA, USA) and 2 mM L-glutamine (Thermo Fischer, Waltham, MA, USA. Connective and adventitia layers were removed, and aortas were transferred to sterile tubes containing collagenase IV (Thermo Fischer, Waltham, MA, USA) (1 mg/mL). After 90 min, the aortas were washed with PBS and the endothelial layer was removed with a sterile cotton swab. Next, the aortas were spread out into culture plates and low-glucose (LG) DMEM (Thermo Fischer, Waltham, MA, USA) supplemented with 10% fetal bovine serum, 40 µg/mL gentamicin sulphate, and 2 mM L-glutamine was added. Plates were maintained at 37 °C with 5% CO_2_. Samples were left undisturbed for 7 days. Isolated VSMCs presented an elongated morphology and stained positively for alpha-actin, a constitutive protein of this type of cell. In addition, homogeneity of VSMC culture was confirmed by flow cytometry. These cells were applied in experimental protocols between passages 5 and 8. Each experiment was performed either twice or three times with three samples (*n* =  6–9). Each cell sample was obtained from a single animal. 

### 4.4. Stimulation and Treatment of Vascular Smooth Muscle Cells

VSMCs were plated on glass coverslips in 24-well plates at a density of 8 × 10^3^ cells/coverslip and allowed to attach at 37 °C under a 5% CO_2_ atmosphere. Serum-starved primary cultured rat VSMCs were challenged with selected concentrations of MT-III (0.2–0.8 μM, dissolved in LG-DMEM) or LG-DMEM alone (control). Where appropriate, the following inhibitors were used: 10 μM GW9662 inhibitor of PPAR-y, 10 μM GSK0660; inhibitor of PPAR-β/δ, 100 nM A922500; inhibitor of DGAT, 100 nM TMP-153; inhibitor of ACAT (Calbiochem-Novabiochem Corp., La Jolla, CA, USA), 250 μM SSO; antagonist of CD36, 250 μM kappa-carrageenan; antagonist of LOX-1, 250 μM fucoidin and dextran sulphate sodium salt (DSF); inhibitors of SR-A1, 100 µM LY294002, 2 μM wortmannin; inhibitors of phosphatidylinositide 3-kinases (PI3K), and inhibitor of endocytosis mediated by receptor, 100 μM dynasore. All stock solutions were prepared in DMSO and stored at −20 °C. Aliquots were diluted in LG-DMEM low glucose to the required concentration immediately before use. The final DMSO concentration was always lower than 1% and had no effect on lipid droplet numbers. All pharmacological inhibitors or < 1% DMSO were added 60 min before the stimulation of VSMCs with MT-III or LG-DMEM (control). Cells treated with the inhibitors were analyzed for viability by the tetrazolium-based (MTT) colorimetric assay. No significant changes in cell viability were registered with any of the above agents or vehicle at the concentrations used.

### 4.5. Lipid Droplet Staining and Quantification

Analysis of lipid droplet numbers was performed in osmium-stained cells. In brief, VSMCs (8 × 10^3^ cells) stimulated with MT-III (0.4 μM) or LG-DMEM (control) adhered to glass coverslips were fixed in 4% paraformaldehyde (PFA) in 0.1 M PBS pH 7.2 for 15 min. The coverslips were then rinsed in 0.1 M phosphate buffer, stained in 1% OsO_4_ (30 min), rinsed in deionized H_2_O, immersed in 1.0% thiocarbohydrazide (5 min), rinsed again in 0.1 M phosphate buffer, restained with 1% OsO_4_ (3 min), rinsed with H_2_O, and then dried and mounted. The morphology of the fixed cells was observed and round osmiophilic structures were identified as lipid droplets, which were then counted by phase-contrast microscopy using a 100× objective lens in 50 consecutively acquired VSMCs in each coverslip.

### 4.6. Western Blotting 

Whole cell extracts were lysed with 100 µL of sample buffer (0.5 M Tris-HCl, pH 6.8, 20% SDS, 1% glycerol, 1 M β-mercaptoethanol, 0.1% bromophenol blue) and boiled for 10 min. Samples were resolved by SDS polyacrylamide gel electrophoresis (SDS-PAGE) on 10% bis-acrylamide gels. Proteins were then transferred to nitrocellulose membranes (GE Healthcare, Buckinghamshire, UK) using a Mini Trans-Blot^®^ (Bio-Rad Laboratories, Richmond, CA, USA). The membranes were blocked for 1 h with 5% non-fat dry milk in TTBS (20 mM Tris, 100 mM NaCl, and 0.5% Tween 20) and incubated with primary antibodies against PLIN2 (Abcam, San Francisco, CA, USA), PLIN3 (Abcam), COX-1 (Merck KGaA, Darmstadt, Germany), COX-2 (Life Technologies Corporation, Carlsbad, CA, USA), CD36 (R&D Systems, Minneapolis, MN, USA), SR-A1 (Thermo Fisher, Waltham, MA, USA) or PPAR-y and β/δ (Santa Cruz, Dallas, TX, USA) or LOX-1, ABCA1, or ABCG1 (Abcam) overnight at 4 °C or β-actin (Sigma-Aldrich Corporation, St. Louis, MO, USA) for 1 h at room temperature. They were then washed and incubated with the appropriate secondary antibodies conjugated to horseradish peroxidase. Detection was performed by the enhanced chemiluminescence (ECL) method according to the manufacturer’s instructions (GE Healthcare). Band densities were quantified with ImageQuant LAS4000 mini (GE Healthcare) mini using the image analysis software from ImageQuant LAS.

### 4.7. Quantification of Phagocytosis

After treatment with non-cytotoxic concentrations of MT-III, VSMCs were exposed to 0.5 μm Flouresbrite^®^ fluorescent beads (Polyscience Inc., Warrington, PA, USA) for 12, 24, and 48 h. Next, cells were washed three times with PBS, and nuclei were stained with Hoechst 33342 fluorescent dye. Data acquisition was accomplished with automated Axiovert 200m (Carl Zeiss MicroImaging GmbH, Jena, Germany) followed by a computerized automatic quantification in the CellProfiler analysis software. 

### 4.8. Quantification of acLDL Uptake

Rat VSMCs were seeded with a density of 10,000 cells per cm^2^ on glass bottom 24-well plates (Zell-Kontakt GmbH, Nörten-Hardenberg, Germany) and cultured overnight in DMEM low glucose supplemented with 10% of fetal bovine serum (Gibco/LifeTechnologies/ThermoFisher scientific; Gibco/Lifetechnologies/ThermoFisher scientific; HyClone SV30079.01 GE). After 24 h, cells were starved for 48 h in DMEM low glucose and stimulated with MT-III (0.4 or 0.8 µM) for 12 h. After stimulation, cells were washed three times with DMEM low glucose and exposed to 1.5 µg/mL AlexaFluor488-labeled acLDL (ThermoFisher Scientific) in DMEM low glucose supplemented with 10% of FCS for 24 h. Cells were then fixed with freshly prepared 3% formaldehyde solution for 10 min. Next, cells were washed three times to remove non-internalized acLDL and stained with Hoechst 33342 (10 µg/mL ThermoFisher scientific). After 60 min, the samples were analyzed by automated microscopy using a Zeiss Axiovert 200m (Carl Zeiss, Jena, Germany) equipped with a motorized stage (Märzhäuser Wetzlar GmbH and Co. KG, Wetzlar, Germany), Colibri2 (Carl Zeiss, Jena, Germany), and the filter set 62HE. Mosaic images with 6 × 8 single images were acquired with a Neofluar 40x/0.75 representing 1000 × 1000 µm of the sample. The images were stitched and exported as Tiff files. The images were scaled down to 50%, resulting in 4000 × 4000 pixel images. High-resolution optical section images of representative areas were created using Apotome2 in conjunction with a Neofluar 40x/1.3 of the Axiovert 200m. The stacks were processed in ImageJ and are shown as maximum intensity projections.

### 4.9. Immunocytochemistry Analysis

Detection of PLIN2, PLIN3, and PPAR-γ and β/δ in MT-III-stimulated VSMCs was performed by immunostaining. Cells were fixed in 2% PFA, permeabilized with 0.2% Triton-X 100 in 0.1 M phosphate buffer, and blocked with 0.5% albumin in 0.1 M phosphate buffer for 30 min. After PBS washes, VSMCs were incubated for 1 h with polyclonal antibodies against PLIN2 and PLIN3 (Abcam, CA, USA) or PPAR-γ and β/δ (1:250) (Santa Cruz, TX, USA) diluted in 0.1 M phosphate buffer with 0.2% Triton-X 100. After three washes with PBS, the preparations were incubated with secondary Alexa488-conjugated antibody (1:500) (Thermo Fisher, Waltham, MA, USA) for 1 h. After the washes, the slides were mounted with Fluoromount-G and examined under a confocal laser scanning microscope (Zeiss LSM 510 Meta). 

### 4.10. Real-Time PCR

To explore the transcriptional changes of the VSMC after MT-III treatment, VSMCs were incubated with LG-DMEM (control) or MT-III (0.4 or 0.8 μM) and lysed with lysis buffer. Total RNA was isolated using the InviTrap^®^ Spin Cell RNA Mini Kit (STRATEC Molecular GmbH). The cDNA was synthesized with the High-Capacity cDNA Reverse Transcription Kit (Thermo Fisher Scientific Inc., Waltham, MA, USA) from 1 mg total RNA using random hexamer priming. Real-time PCR was performed to detect the relative expression levels of genes known to describe the phenotypic shift, such as MAC-2, CD68, and CD163 (to characterize macrophage-like phenotype), as well as smooth muscle actin, SM22, and myosin heavy chain (to characterize VSMCs)^44^ normalized to HPRT-1 using the CFX96 real-time PCR System (Bio-Rad Laboratories) and SYBR Premix ExTaq™ (Lonza Group Ltd., Basel, Switzerland). 

### 4.11. Statistical Analysis 

Data are expressed as the mean ± standard error of the mean (SEM) of at least three independent experiments. Multiple comparisons among groups were performed by one-way analysis of variance (ANOVA) followed by Tukey’s test. Values of probability lower than 5% (*p* < 0.05) were considered significant.
